# The role of gold weight implants in the management of paralytic lagophthalmos

**DOI:** 10.3906/sag-2104-50

**Published:** 2021-10-21

**Authors:** Muammer Melih ŞAHİN, Eray UZUNOĞLU, Recep KARAMERT, Süleyman CEBECİ, Gökçen CESUR, Mücahit YALÇIN, Mehmet DÜZLÜ, Hakan TUTAR, Mehmet Birol UĞUR, Alper CEYLAN

**Affiliations:** 1 Department of Otorhinolaryngology/Head and Neck Surgery, Faculty of Medicine, Gazi University, Ankara Turkey

**Keywords:** Gold weight implant, facial nerve paralysis, facial nerve, facial reanimation, acoustic neuroma

## Abstract

**Background/aim:**

The study aims to evaluate the usage of gold weight implants and monitor complaints and comfort of patients.

**Materials and methods:**

A hundred and ninety-one implantations performed between January 2009 and January 2019 were analyzed. Seventy-eight patients included in this study The average age of the patients was 51.3 ± 14.5 years. Forty-five (57.7%) of them were male and 33 (42.3%) female. Patient satisfaction was measured with a questionnaire containing the most common complaints related to gold weight in the literature through telephone surveys.

**Results:**

The average follow-up time was 74.5 months. Ninety-three-point-five percent of subjects had operational causes, among which the most widespread was acoustic neuroma (44.9%). The average time between facial paralysis and implantation was 141.1 days. Implantation was performed 26.6 days on average after acoustic neuroma surgery and 3.2 days on average after temporal zone malignancy surgery. Thirty-eight patients had their implants removed over either complication (n = 14) or recovery (n = 24). Recovery was the fastest after facial nerve decompression (mean= 4.75 ± 3.6 (2–10) months) and the slowest after 7–12 cranial nerve transfer (mean= 18.3 ± 8.2 (3–31) months). Twenty-six-point-nine percent (n = 21) of patients had complications, of which the most common was extrusion (n = 10). The overall satisfaction rate was 88.5% with the highest in visual acuity and the lowest in continuous requirement for artificial tear.

**Conclusion:**

The gold weight implantation is an effective, reversible, and easy procedure significantly reducing complaints regarding paralytic lagophthalmos. Early implementation may be beneficial for ocular complications. A dynamic facial reanimation could terminate need of implant.

## 1. Introduction 

Advanced peripheral facial paralysis that affects eye closure function is frequently seen after otologic, neurotologic, and lateral skull base surgeries [1]. In addition, cholesteatoma, some malignant tumors, and idiopathic facial paralysis (Bell’s palsy) can cause loss of eye closure function [2]. The reason for the loss of eye closure function is the loss of innervation and function of the orbicularis oculi muscle. Moreover, the loss of parasympathetic innervation of lacrimal gland disturbs production of tear and the loss of eyelid tone may lead to eversion of the eyelid. On the other hand, levator palpebrae superior muscle that is still functioning ensures the eye to remain open. This condition is called lagophthalmos and accompanied by poor lid closure, incomplete blinking, lower eyelid ectropion, and eye dry out. This could lead to keratitis and corneal abrasion, followed by loss of vision, and eventually blindness [1,3]. Management of lagophthalmos caused by facial paralysis includes various surgical and non-surgical methods. Eye-patching and lubricant drops could be beneficial when spontaneous recovery is anticipated in the short term. In case there is permanent damage to facial nerve or no expectation of a quick recovery, surgical management should be employed. Surgical managements are grouped as dynamic and static procedures. Dynamic procedures involve nerve repair and muscle transposition whereas static procedures consist of temporary or permanent tarsorrhaphy, mullerectomy, and lid loading. Lid loading with gold weight is an effective and widely-used method [4]. Its low complication rates, easy application, and reversibility have contributed to the popularity of the method. Gold is the most preferred material in lid loading due to its nonallergic properties, malleability, high density, and color match for the skin [5]. Thanks to inert nature of gold, allergy has been reported in only one case [6].

Even if blink reflex could not be reestablished, voluntary closure of eyelid could be restored. The method depends on the gravitational action of the weight against force vector of levator palpebrae superioris muscle. Since it is a mechanical method rather than being a physiological procedure, situations such as sense of foreign body, eye dryness, blurred vision, and extrusion of the implant that negatively affect the quality of life may occur in the long term. The aim of the study is to evaluate the usage of gold weight implants and monitor the complaints and comfort of patients.

## 2. Patients and method

A hundred and ninety-one gold weight implantations performed between January 2009 and January 2019 at our otorhinolaryngology clinic were analyzed retrospectively. In addition to the patients who had passed away by the time of the study, those who had undergone the following operations were excluded: a previous eyelid surgery such as blepharoplasty and levator resection that may have caused a scar in the upper lid, a revision of gold weight implantation. Patients with inadequate follow-up data were also excluded. Ninety-one patients who met the criteria were interviewed by phone. Seventy-eight patients who answered the questionnaire were included in this study.

Patients’ demographic data, causes of facial paralysis, durations of gold weight usage, causes of extraction of implant, and complications encountered were obtained from patient files. To evaluate the factors that could influence the overall satisfaction, we conducted a questionnaire containing the most common complaints related to gold weight in the literature [7–10]. The patient satisfaction questionnaire was conducted by phone call. The questions answered by the voluntary patients are shown in Table 1. For each question (symptom), patients chose one of the following answers: highly dissatisfied, dissatisfied, normal, satisfied, and highly satisfied. Moreover, patients were asked about overall satisfaction. The study was approved by the local ethical committee of Gazi University (91610558-604.01.02-). Statistical analysis was performed through IBM SPSS Statistics v: 22.0 (IBM Corp., Armonk, NY, USA). Kaplan–Meier survival estimate was conducted for extrusion and recovery. Descriptive data were presented as number (n) and percentage (%).

**Table 1:** Questionnaire of patients’ perspectives on ocular symptoms.

	Level of satisfaction				
Questions (Symptoms)	Highly dissatisfied (%)	Dissatisfied (%)	Normal (%)	Satisfied (%)	Highly satisfied (%)
Absence of pain	1.3	1.3	5.1	21.8	70.5
Absence of tearing	-	5.1	7.7	32.1	55.1
Absence of redness	1.3	1.3	9.0	28.2	60.3
Absence of foreign body sensation	-	3.8	7.7	21.8	66.7
Visual acuity	1.3	-	3.8	15.4	79.5
Absence of need for artificial tear	7.7	10.3	38.5	33.3	10.3
Daytime eye closing ability	-	1.3	11.5	35.9	51.3
In-sleep eye closing ability	-	3.8	10.3	34.6	51.3
Aesthetical appearance	1.3	7.7	9.0	42.3	39.7

### 2.1. Surgical method

Initially, the proper weight of the gold planned to be implanted was measured meticulously. To determine the ideal weight, each patient sat in an upright position and weights ranging from 1.2 g to 2.2 g were incrementally placed on upper lid till sufficient eye closure was acquired. All surgeries were performed by an otorhinolaryngologist. All of the procedures were implemented under local anesthesia and 1½ cm incision was made on upper eyelid crease. After skin incision, orbicularis oculi muscle was dissected and a pocket 2 mm above the upper eyelash margin was created between orbicularis oculi muscle and tarsal plate. A gold weight, curved according to the shape of eye globe with two holes on the corners and one on the opposite edge, was inserted into the pocket. The gold weight was fixed to the tarsal plate with 6.0 prolene suture (Ethicon US, LLC). Orbicularis oculi muscle and skin incisions were sutured respectively with 5.0 monocryl suture (Ethicon US LLC). A dressing was made with sterile strips. All the patients were discharged on the same day as the operation. 

## 3. Results

The average age of the patients was 51.3 ± 14.5 years. Forty-five (57.7%) of them were male and 33 (42.3%) female (Table 2). The average follow-up time was 74.5 months. The causes of facial paralysis were shown in Table 3. Surgery was the main cause of paralysis and comprised 93.5% of the eyelid loading indications. The average time between facial paralysis and gold weight implantation was 147.1 days. The average intervals for each indication were shown in Table 3. 

**Table 2:** Demographics and time intervals.

General information	
Age	51.3 ± 14.5 years
Sex	
Male	45 (57.7%)
Female	33 (42.3%)
Side	
Right	42 (54%)
Left	36 (46%)
Status	
Present	40 (51.3%)
Complication related removal	14 (18%)
Recovery related removal	24 (30.7%)
Interval between FP-GWI	147.1 ± 817.7 days (2–7200)
Interval between GWI-removal	16.0 ± 13.2 (1–72) months
Complication related	18.3 ± 18.8 (1–72) months
Recovery related	14.6 ± 8.7 (2–31) months
Ongoing usage time	69.6 ± 29.0 (15–131) months
Follow-up time	74.5 months

GWI: Gold weight implantation, FP: Facial paralysis.

**Table 3:** Causes of lagophthalmos and durations between facial paralysis and gold weight implantation.

Indications	n	%	Time(day)
Acoustic neuroma	35	44.9	26.6
Cholesteatoma	11	14.1	274.2
Temporal zone malignity	6	7.7	3.2
Parotid gland malignity	5	6.4	88.3
Mastoid bone surgery	4	5.1	58.7
Malignant pontocerebellar angle tumor	4	5.1	37.5
Traumatic temporal bone fracture	3	3.8	178.6
Idiopathic peripheral facial palsy	2	2.6	~
Facial Schwannoma	2	2.6	27.0
Meningioma	2	2.6	106.5
Glomus Jugulotympanicum	1	1.3	30.0
Chordoma	1	1.3	32
Neurofibroma	1	1.3	30
Pons cavernous hemangioma	1	1.3	54
TOTAL	78	100	

**Figure 1:**
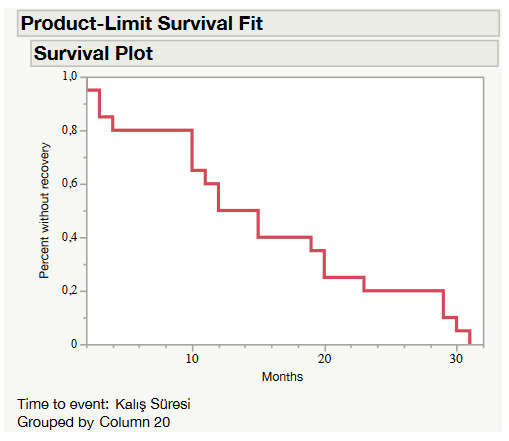
Kaplan–Meier survival plot for subjects whose gold weight implant was removed after a successful facial reanimation procedure

The gold weight implant of 38 patients had been removed by the time of the questionnaire (Table 4). Fourteen of these removal procedures had resulted from complications such as extrusion (n = 10), migration (n = 2), and infection (n = 2). Twenty-four patients had their implants removed over recovery (either spontaneous or through facial reanimation). Four of these removals had been caused by spontaneous recovery of facial nerve function. The remaining 20 patients had undergone a successful facial reanimation procedure before removal: thirteen of them had had 7–12 cranial nerve transfer surgery, four facial nerve decompression surgery, and three facial nerve repair with cable graft. For all 38 patients, the average duration between the implantation and the removal was 16.0 ± 13.2 months (1–72). Durations concerning removal by complication and removal by recovery were 18.3 ± 18.8 (1–72) and 14.6 ± 8.7 (2–31) months, respectively and the difference was not statistically significant. This figure was 18.3 ± 8.2 (3–31) months for 7–12 cranial nerve transfer surgery, 17.0 ± 11.2 (10–30) months for cable graft, 4.75 ± 3.6 (2–10) months for facial nerve decompression, and 11.0 ± 1.6 (9–13) months for spontaneous recovery. A Kaplan–Meier survival estimate conducted with the patient who had their implants removed due to a successful facial reanimation procedure showed that half of the removals due to recovery occurred in a year after implantation (Figure 1). 

**Table 4:** Causes of removal of the gold weight implants.

Cause of removal	n	%
7–12 Anastomosis	13	34.2
Extrusion	10	26.3
Spontaneous gain of function	4	10.5
Decompression of facial nerve	4	10.5
Facial nerve repair with cable graft	3	7.9
Migration	2	5.3
Infection	2	5.3
TOTAL	38	100

**Figure 2:**
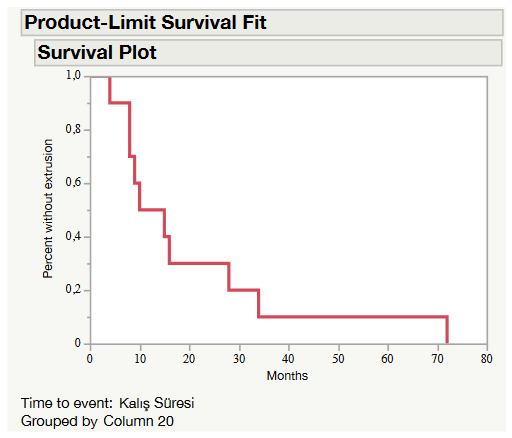
Kaplan–Meier survival plot for subjects who complicated with the extrusion of the gold weight implant

A complication occurred in 26.9% (n = 21) of the patients (Table 5). Extrusion of the implant was the most common complication (n = 10). The average time of extrusion was 20.4 ± 20.4 (4–72) months. Kaplan–Meier survival estimates conducted with patients who were complicated with extrusion of the gold weight showed that about 80% of this complication occurred within two years after the implantation (Figure 2). The other complications were dislocation and migration of the implant (5.1%, n = 4), infection (5.1%, n = 4), residual lagophthalmos (2.6%, n = 2), and ectropion of the upper eyelid (1.3%, n = 1). 

**Table 5:** Complications.

Complications	n	%
Migration	4	5.1
Infection	4	5.1
Extrusion	10	12.8
Residual Lagophthalmos	2	2.6
Ectropion/Entropion	1	1.3
TOTAL	21	26.9

The results of the patient satisfaction questionnaire were shown in Table 1. Forty patients had their implants in situ at the time of the questionnaire. Visual acuity and pain were the symptoms that received the highest rates of satisfaction: 70.5% of the patients were highly satisfied with the absence of pain while 79.5% of them were highly satisfied with their visual acuity. Artificial tear usage had the lowest complacency rate: Only 10.3% of the patients were highly satisfied with the absence of need for artificial tear whereas 7.3% of them were highly dissatisfied with needing to use it. 9% of the patients were not pleased with aesthetical appearance of the implant. When the patients were asked about their gladness of gold weight implant broadly, 88.5% of them (n = 69) declared contentment. 

## 4. Discussion

The present study investigated the role of lid loading with gold weight in the management of paralytic lagophthalmos and assessed patients’ perspectives on ocular symptoms. Although lid loading methods provide voluntary eye closure with the help of gravity, blinking reflex could not be reestablished. Even if these methods do not offer a physiological recovery, high rates of success and satisfaction have been presented in the literature [11]. Kelley et al. reported that 72% of patients in their study felt more comfortable after lid loading [12]. Misra et al. noted that the majority of the participants (88%) stated improvement [9]. In a study by May, which is one of the largest series in the literature, a success rate of 90% was observed [11]. Rofagha reported that 97% of the operations of eyelid loading were successful in providing improved protection for the patient’s eye and decreasing daily care needs [13]. In the present study, the overall satisfaction rate was 88.5% and this outcome was consistent with literature data. However, it is not easy to access detailed information in the above-mentioned studies that may affect contentedness independently. The patients were asked about the complaints of pain, tearing, redness, sense of foreign body, loss of visual acuity, artificial tear usage, and the lack of eye closing ability during daytime and sleep as well as aesthetical appearance. They chose one of the following answers for each question (symptom): highly dissatisfied, dissatisfied, normal, satisfied, and highly satisfied. 

In our questionnaire, we found the highest satisfaction in visual acuity. Sonmez et al. claim that the visual acuity is related to the degree of pain and tearing [7]. In agreement with that argument, we think that the high rate of satisfaction regarding the visual acuity in our study is associated with the high satisfaction rates in the complaints of pain and tearing. For the absence of pain, tearing, and redness, pleasantness (satisfied or highly satisfied) was expressed by 92.3%, 87.2%, and 88.5% of the patients, respectively. Moreover, no patients suffered loss of visual acuity in the present study. Pickford reported loss of vision in 15% of the patients with high rates of tearing and soreness complaints [10]. In our practice, when the facial nerve damage was permanent or no quick recovery was expected after a surgery, we performed the gold weight implantation in the early postoperative period before discharge. Sharon et al. reported that 25% of the corneal complications occurred in 18 days and 50% occurred in 2 months after lateral skull base surgery and recommended early administration of lid loading procedures, preferably during hospital stay [1]. The low rates of pain, redness, and tearing in the present study may be related to this principle. For instance, gold weight implantation was performed 26.6 days on average after acoustic neuroma surgery and 3.2 days on average after temporal zone malignancy surgery. 

In our study, 1.3% of the patients were highly dissatisfied and 7.7% were dissatisfied with cosmetic appearance and the main reason was the visibility of the implant. Rofagha reported the rate of cosmetic dissatisfaction to be 5% in her study and claimed that this was related to placing the implant too low near the lid margin [13]. However, to provide eye closing in supine position we preferred to place the implant close to the lid margin. In our study, only 3.8% of the patients were not pleased with eye closing ability during sleep. Placing the implant low with respect to the lid margin harms the aesthetic appearance. On the other hand, a superiorly placed implant needs to be heavier to provide the same gravitational force [7]. For aesthetic appearance, the rate of highly satisfied or satisfied patients was 82% in the present study. Pickford et al. assessed the effects of lid loading on overall facial appearance and noted that 60% of the patients stated the appearance was improved [10].

In our study, the most dissatisfying issue was the necessity of artificial tear usage on a continuous basis. In our clinical practice, we strictly recommend using artificial tears every two hours during the day even if a proper voluntary eye closure is obtained. The disturbance of tear production and loss of blinking reflex are still major triggers for corneal complications. As Sharon et al. stated, intensive corneal lubrication regimen is the single most important preventive strategy for maintaining corneal integrity [1].

Lavy et al. observed that foreign body sensation may occur even after a successful implantation procedure [14]. The dissatisfaction rate concerning the foreign body sensation was also low in our study and none of the patients requested removal of the gold weight because of this complaint. Using an implant with a curve that matches the shape of the eye globe may reduce foreign body sensation. The flexible implants may be an option to provide a more suitable shape, but their usefulness is controversial in the literature. A recent study reported that flexible implants had a higher extrusion rate compared to that of rigid implants [15]. On the other hand, Braun et al. reported a low complication rate, high satisfaction level, and improvement in the quality of life with the platinum chain implants [16].

Similar to other questionnaire studies, some patients did not have an implant at the time of the phone call because it had already been removed due to complications or recovery [9,10]. While 15.7% of the patients had no implant in the questionnaire study by Mısra et al., this rate was 48.7% (n = 38) at the time of the phone call in our study. Our clinic has a neuro-otology department and we often implement a dynamic facial reanimation procedure for patients, especially for those with an operative cause. In our study, 52.6% of the removal operations were carried out after employment of a successful facial reanimation procedure such as 7–12 anastomosis (n = 13), decompression (n = 4), and nerve repair with cable graft (n = 3). After facial nerve decompression, recovery was the fastest with a mean time of 4.75 ± 3.6 (2–10) months. As expected, it took longer with 18.3 ± 8.2 (3–31) months on average to obtain a voluntary eye closure after 7–12 cranial nerve transfer. More than half of the removals due to recovery occurred within one year. 

As reported in the literature, surgery is the main indication for eyelid loading in the majority of patients. In a study including 76 patients, 46% of subjects underwent acoustic neuroma surgery and 26% had other surgeries [9]. In the present study, 93.5% of subjects had an operational cause and the most common one was acoustic neuroma with 44.9%. Thus, in an attempt to control the main pathologies during follow-up, magnetic resonance imaging (MRI) is important for these patients who had facial palsy as a consequence of tumors. The presence of the implant is not contraindicated to MRI [17,18]. We did not encounter a problem with MRI in our study. 

Aggarwal et al. excluded in their study the patients who had prior gold weight implantation or lid surgery [19]. Rofagha and Pickford claimed that previous eyelid surgery increases the risk of complications [10,13]. In a comprehensive satisfaction questionnaire study involving 76 patients, subjects with previous surgeries and revisions were not excluded [9]. We excluded the patients with a previous surgery that may have caused a scar in the upper lid, resulting in a total number of 78 patients in the present study. Consistent with the literature, the rate of complication was 26.9% in our study [7,14,20]. Kelley et al. observed a higher complication rate of 68% and claimed that extrusion of the prosthesis (43%) was the most prevalent complication that often occurred a long time after insertion (follow-up time: 18 years) [12]. The rates of extrusion presented in the literature range between 0% and 43% [3,12,14,21,22]. Many studies reported lower extrusion rates within shorter follow-up periods. Extrusion was also the most common complication in our study and the rate was 12.8%. Follow-up time was 74.5 months and the average duration for the extrusion of the implant was 20.4 ± 20.4 (4–72) months in our study. Moreover, nearly 80% of this complication occurred within 2 years. Kelley et al. claim that the risk of extrusion is reduced by choosing the smallest weight necessary, placing it deeply through a medial incision, and fixing it to tarsal plate and recommend that in case of extrusion, it may be removed and replaced with a smaller weight [12]. The migration rate in our study was 5.1%. Misra et al. observed medial shift of the gold weight in 15 of 76 patients (19.7%) [9]. In the literature, it is suggested as the safest and optimal method to fix the implant to the tarsal plate after transverse supratarsal incision [7,9,10,12]. Since the fixation procedure was applied to all patients included in our study, it was not possible to assess whether or not the fixation was protected from complications, especially from migration. Infection is another complication that entails serious consideration. Antibiotic treatment should be employed in case of an infection but even after a proper antibiotherapy, removal of the implant may be required. Furthermore, if a noninfectious inflammatory response to implant is suspected, corticosteroids could be administered but removal of the implant still remains an option [23,24]. In our case series, we removed two implants due to infection nonresponsive to the antibiotics. We did not encounter any hypersensitivity reaction to the implants in the patients. Residual lagophthalmos and ptosis are the recognized complications of this method and avoidance these complications depends on accurate estimation of the weight [3,9]. The rates of these complications reported in the literature range between 2%–30% [14, 19,20,25,26]. We carefully measured the optimal gold weight in all patients; thus these complications were quite rare in the present study. We encountered residual lagophthalmos in only two (2.6%) patients. 

The gold weight implantation is an effective, satisfactory, reversible, and easily applied procedure and it significantly reduces expected complaints related to paralytic lagophthalmos. Almost all of the requirements for eyelid loading stem from a surgical procedure and the most common one is acoustic neuroma surgery. Implementation of the implant as early as possible may be more beneficial to protect the patient from ocular complications. Applying an intensive corneal lubrication regimen was the most common complaint of patients in our study. It is noteworthy that the implant was removed in almost half of the patients. For the vast majority of these patients, the reason was that they had undergone a dynamic facial nerve recovery operation that eliminated weight use. The other reason was complications. The most commonly performed recovery technique was the 7–12 cranial nerve transfer and the most widespread complication was extrusion. The complication rates increased with long follow-up periods. 

## Informed Consent

The study was approved by the local ethical committee of Gazi University (91610558-604.01.02-). All participants provided informed consent.
